# Foreshadowing of Performance Accuracy by Event-Related Potentials: Evidence from a Minimal-Conflict Task

**DOI:** 10.1371/journal.pone.0038006

**Published:** 2012-05-31

**Authors:** Hiroaki Masaki, Timothy I. Murphy, Keita Kamijo, Katuo Yamazaki, Werner Sommer

**Affiliations:** 1 Faculty of Sport Sciences, Waseda University, Tokorozawa, Saitama, Japan; 2 Department of Psychology, Brock University, St. Catharines, Ontario, Canada; 3 Institut für Psychologie, Humboldt-Universität zu Berlin, Berlin, Germany; University Medical Center Groningen UMCG, Netherlands

## Abstract

**Background:**

Recent studies employing stimulus-response compatibility tasks suggest that an increase in the amplitude of the positive deflection of the response-locked event-related potential (ERP) foreshadows errors on forthcoming trials. However, no studies have tested the generalizability of error-foreshadowing positivity to tasks without stimulus-response interference.

**Methodology/Principal Findings:**

The present study adopted an alternating-response task, in which the participants responded to the pointing direction of an arrowhead (up or down). Although the arrowhead direction alternated for the majority of trials (95%), occasionally this pattern was broken by a repeated stimulus, termed a lure trial. We compared the matched-reaction-time correct-preceding ERP with the error-preceding ERP on lure-preceding trials. There was no evidence that errors are foreshadowed by the increase of a positive electroencephalogram (EEG) deflection. To the contrary, analyses of ERPs time-locked to electromyogram (EMG) onset on the five consecutive lure-preceding trials showed larger positive deflections on correct-preceding than error-preceding trials. The post-response negativity did not differ between correct-preceding and error-preceding trials.

**Conclusions/Significance:**

These results suggest that in minimal conflict tasks a decreased positivity may foreshadow incorrect performance several trials prior to the error, possibly reflecting the waning of task-related efforts. Therefore, error-foreshadowing brain signals may be task-specific.

## Introduction

If a specific brain activity foreshadows performance accuracy, it may be a useful signal to prevent individuals from making mistakes in various kinds of tasks. Some studies have found that motor-related activity may foreshadow or predict performance accuracy. For example, a larger Bereitschaftspotential prior to a self-paced button press initiating a motor task foreshadowed better performance [1]. In a time discrimination task, cortical activity over prefrontal areas decreases prior to correct performance, reflecting efficient temporal processing [2]. Recent studies have suggested that event-related potentials (ERPs) associated with performance monitoring can foreshadow erroneous responses in cognitive conflict tasks, as explained below.

Performance monitoring is seen to be reflected in a negative component that can be elicited by incorrect responses (errors, the error-related negativity: ERN), as well as correct responses (the correct-response negativity: CRN) [3]. The ERN and CRN are similar in terms of latency, topography, and functional relationships [4]. The ERN has a frontocentral distribution, presumably reflecting neural activity of the anterior cingulate cortex (ACC), and is thought by some researchers to be functionally related to error-detection [5], [6] or by others to be related to detection of response conflicts [7], arising from the crosstalk interference that occurs when two response activations overlap during the parallel processing of incongruent stimuli [8]. Therefore, it has also been suggested that the CRN is involved in performance monitoring [9]. On the other hand, the CRN may also be due to stimulus-related ERP activity or contamination of an ERN elicited by partial (incomplete) errors [10], [11].

On the assumption that the CRN signals performance monitoring, transient variations in the efficiency of the monitoring system should be reflected in amplitude changes of the CRN and in variations of the error rates in subsequent trials. Indeed, Ridderinkhof, Nieuwenhuis, and Bashore [12] hypothesized that trial-to-trial fluctuations in the ERP amplitude reflect variations in the efficiency of performance monitoring, and thus could represent the relative efficiency of executive control. They compared ERPs on correct trials preceding error responses (error-preceding) with correct trials preceding correct responses (correct-preceding). They found an enhanced positive deflection peaking approximately 50 ms at a fronto-central electrode (FCz) after the button-press response in error-preceding trials and, referred to it as error-preceding positivity (EPP). These findings were replicated in subsequent studies. Allain, Carbonnell, Falkenstein, Burle, and Vidal [13] reported a less negative CRN (hence more positivity) on the trials preceding an error response. Hajcak, Nieuwenhuis, Ridderinkoff, and Simons [14] found that negativity was not reduced on the error-2 trial and suggested that the disengagement of the response-monitoring system is specific to the error-1 trial in the Eriksen flanker task [15].

As mentioned above, some researchers believe that the negativity observed after errors could be elicited by the response conflict due to a competitive process between correct and incorrect response activations. This conflict may arise on any trial in which an error is initiated, even if the task itself was not designed to produce conflict. Thus, response conflict may be one critical factor in determining the size of the negativity. Previous studies that investigated brain activity on error-preceding trials matched these with correct trials in terms of reaction time (RT) to reduce the differences in response conflict, but did not report if ERPs were separately averaged for error-preceding congruent and incongruent trials [12], [13], [14]. This may be important for two reasons.

First, Gratton, Coles, and Donchin [16] as well as Stürmer, Leuthold, Soetens, Schröter, and Sommer [17] clearly showed stronger interference effects following congruent than incongruent trials in the Eriksen flanker and in the Simon task [18], respectively; thus, errors are more likely after congruent trials (see also [19]). Because the response-conflict account predicts larger negativity for correct responses on incongruent trials than on congruent trials (e.g., [7]), it is plausible to hypothesize that the larger positivity on the error-preceding trial (i.e., error-1 trial) that previous reports emphasized, was partly due to the relatively small number of incongruent trials included in the ERP averaging on those trials. Thus, the assertion that the negative component observed on the correct-preceding trial is associated with the inhibition process of erroneous response on the following trial may be challenged.

Second, previous studies that reported the EPP used some type of response-conflict task. According to previous findings outlined above [16], [17], it is to be expected that preceding an error there is likely a smaller proportion of incongruent trials than preceding a correct trial. It is known that incongruent trials elicit a CRN and therefore the relative positivity preceding an error could be due to fewer incongruent trials eliciting CRN responses. It seems that few studies of the EPP have controlled for the proportion of incompatible and compatible trials preceding errors and correct trials.

Taking these factors into account, response-conflict tasks (e.g, the flanker task) may involve rather specific ERP components and performance-foreshadowing phenomena. Therefore it seems to be important to use a variety of other tasks not involving conflict in order to identify task-specific and task-general error-preceding activities. In the present study, we investigated if ERPs could foreshadow the subsequent performance in a task that differed from previously published studies in terms of response-conflict. It would provide a promising tool for human-error research if the EPP could be shown to be a general component that can also be observed in tasks that do not involve response conflict.

To avoid the issue of response-conflict we adopted an alternating-response task, in which the participants responded compatibly to up- or down-ward pointing arrow heads with the left or right hand placed on buttons in the midsaggital line. In the majority of trials responses alternated between the left and right hand but occasionally a response had to be repeated. These repetition trials will be referred to as *lure* trials. Because the arrow-head presented on the monitor simply indicates the responding direction, there should be virtually no response-conflict in the lure-*preceding* trials. This type of task has been used in sport psychological studies in terms of the anticipation process associated with feinting stimulus (e.g., [20]). We recorded the electromyogram (EMG) to obtain a more precise measure of response activation and to exclude any partial errors where the participant initiates an erroneous response, but does not make an overt error. It is likely that the lure stimulus should induce response-conflict between the preponderant response tendency to alternate and the need to repeat the response. However, there should be no such conflicts preceding the alternations trials.

In the present study, we investigated brain activity on the five trials immediately preceding the lure trial. Previous studies [12], [14] adopted a matched RT procedure to rule out the possibility that the EPP reflects stimulus-synchronized activities. To this end, they compared ERPs for the error-preceding trial and for the correct-preceding trial using an equal number of trials of each type that had been matched based on RT. We agree that the RT-matching procedure may be important to clarify the brain activity preceding errors. In this study, we also compared ERPs on the lure-1 trial across error-preceding, correct-preceding, and RT-matched correct-preceding cases. On the other hand, the RT-matching procedure makes it impossible to investigate gradually changing efficiency of response monitoring in the trials preceding the lure trial. Thus, we also directly compared each combination of correct- and error-preceding trial to clarify the processes that would be changed as a function of the time course preceding the lure trials. If the impairment of response monitoring occurs on several trials preceding the lure trial, such deterioration should be represented as changes in ERP components. Alternatively, if the improvement of response monitoring transiently occurs on several trials preceding the lure trial, such beneficial processing should also be represented as changes in ERP components. Therefore, we focussed our analyses to brain processing on the lure-preceding trials, although ERPs were averaged for correct and error responses on the lure trial and the post-lure trial, respectively.

It was the main aim of the present study to investigate whether previous findings regarding error preceding brain activity in conflict tasks would generalize to a task that involves minimal conflicts. If an impairment of response monitoring is error predictive even in our task, smaller negativities and/or larger EPPs on error-preceding trials would be obtained. Conversely, if the negativity representing response monitoring would be small or absent on the error-1 trial due to the absence of incongruent trials we would not expect any EPP on the error-1 trials. In any case, because the task employed in the present study was different from those previously used in error prediction research, one might see completely different or unique ERP phenomena predicting the error.

## Results

### Reaction Time

**Figure 1:**
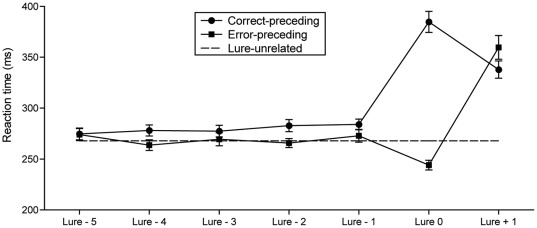
Reaction time in a series of five consecutive trials. Error bars represent *SEM*. A horizontal dotted line in the figure indicates the overall RT.

Figure 1 shows mean RT on a series of five consecutive trials relative to the lure trial. The horizontal dotted-line in the figure represents mean overall RT (*M* = 267.9 ms, *SEM* = 6.18 ms) of the correct-following trials (excluding all trials from lure-5 to lure+1). On the lure trial, a clear bifurcated direction of RT relative to the lure-1 trial was observed. The RT was longer on correct responses and shorter on incorrect responses. On the lure+1 trial, prominent post-error slowing relative to the overall RT (*M* = 91.7 ms, *SEM* = 8.15 ms) was observed following an error response. For the lure preceding trials, RT appeared to be slightly faster on error-preceding than on correct-preceding trials. This effect was observed even on the lure-3 trial.

A two (correct/error) by seven (trial lure-5 through lure+1) ANOVA on RT revealed main effects of correctness (*F*(1, 18) = 90.49, *p*<.001, *pη^2^* = .83) and trial (*F*(6, 108) = 105.53, *ε* = .35, *p*<.001, *pη^2^* = .85). An interaction of correctness and trial was also significant (*F*(6, 108) = 110.30, *ε* = .55, *p*<.001, *pη^2^* = .86), supporting the observation mentioned above. Simple effects tests confirmed significantly longer RTs on correct versus error-related responses on trials lure-1 (*p* = .007), lure-2 (*p*<.001), and lure-4 (*p*<.001). These tests were uncorrected; however, even with a Bonferroni correction for 7 comparisons (*p*<.007 required to claim significance) these would all remain significant. The other two comparisons were in the expected direction but only the lure-3 trial was marginally significant (*p* = .12), while the lure-5 trial showed no significant difference (*p* = .86). In addition, correct responses had a significantly slower RT (*p*<.001) and erroneous responses showed significantly faster RT (*p*<.001) on the lure trial relative to the lure-1 trial. Both post-error slowing and post-correct speed-up relative to the lure trial were also statistically supported (both *p*<.001). Notebaert, Houtman, Van Opstal, Gevers, Fias, and Verguts [21] suggested that the post-error slowing that is typically observed may be due, in part to the orienting effect of an unexpected stimulus so we also compared the difference between the lure+1 and lure−1 RT for both correct and erroneous responses to the lure. There was a greater degree of slowing from pre-error to post-error (86.9 ms) than pre-correct to post-correct (53.8 ms) trials, *t*(18) = 3.46, *p* = .003. Thus, the assertion of Notebaert et al. [21] does not appear to be fully supported here because the errors produced a larger increase in RT than did a correct response.

In addition, we compared mean RTs for the 5 correct-preceding (279.4 ms) and 5 error-preceding trials (269.1 ms), and overall RT (all other remaining trials, 267.9 ms). One-way ANOVA revealed significant differences (*F*(2, 36) = 11.39, *ε* = .87, *p*<.001, *pη* ^2^ = .39), showing a longer RT for correct-preceding than RT for error-preceding (*p*<.001) and overall RT (*p*<.001). There was no difference between RT for error-preceding trials and the overall RT (*p* = .70).

Mean error rate on the lure trial was 50.5% (*SEM* = 2.56%), and overall mean error rate was 4.7% (*SEM* = 0.29%). Mean rate of no response was 0.9% (*SEM* = 0.25%). We also tested that errors might be distributed differently across the experiment by comparing the error rate in the first versus second half of the task (*M* = 47.6% vs. 53.1%); however this difference was not statistically significant (*p* = .38).

### ERPs

**Figure 2:**
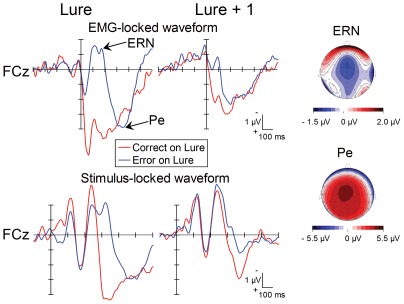
The grand-averaged waveforms of the EMG-locked ERPs at FCz associated with correct (red) and error responses (blue) on lure trials (left panel) and following correct and incorrect trials (lure+1) (right panel). Waveforms are drawn with negative polarity up. Topographies of the ERN (ranging from 156 to 188 ms after EMG onset) and Pe (340 to 371 ms after EMG onset) for error responses on the lure trial are also shown (spherical spline interpolation of order 4, with maximum degree of Legendre Polynomials of 10).

Figure 2 (left panel) depicts both the EMG-locked and the stimulus-locked grand-averaged ERPs at FCz on the lure trials. When the participants did not inhibit and correct an initially erroneous response, the ERN occurred, peaking around 150 ms after the erroneous EMG onset. The ERN was followed by an error positivity (Pe) [5], peaking approximately 350 ms after the EMG onset. On the other hand, no Pe was observed on trials with correct responses, although a CRN appears to have been produced (see the EMG-locked ERPs in Figure 2). However, it has been argued that the CRN may be an artifact of the stimulus-locked N2 superimposed on the EMG-locked ERP [10]. Given the similarity of the latencies in this case (∼250 ms after stimulus) we feel that this account may also explain the present negativity in correct response-locked ERPs.

The stimulus-locked ERPs showed a larger negativity peaking about 250 ms that was followed by a large positive deflection ranging from 350 to 600 ms after the stimulus onset on correct trials. The positive deflection showed a centroparietal distribution.

Figure 2 (right panel) depicts the grand-averaged ERPs on the lure+1 trial. The post-EMG negativity peaking about 100 ms after the EMG onset appears to be much more positive on the correct-following trials; however, this is presumably due to contamination of the stimulus-synchronized activities (see the EMG-locked ERP). The stimulus-locked ERPs showed a frontocentrally distributed positivity that followed the post-EMG negativity.

**Figure 3:**
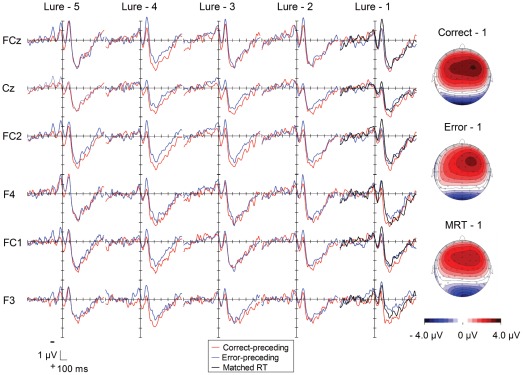
Grand-average waveforms of EMG-locked ERPs as a function of lure-preceding trials (red: correct-preceding, blue: error-preceding trials). Waveforms are drawn with negative polarity up. Scalp distributions of the positivity following the response (ranging from 199 to 230 ms) are shown for the lure - 1 trials (i.e., correct-preceding, error-preceding, and matched RT trials).

Figure 3 shows the EMG-locked ERPs over frontocentral regions (i.e., F3, F4, FC1, FC2, FCz and Cz) on the lure-preceding trials. The EMG-locked ERPs show a negative deflection peaking about 100 ms after the EMG onset, which was followed by a positive deflection peaking about 210 ms after the EMG onset. The negative deflection showed a more frontal distribution, and the positive deflection showed a more broadly and slightly right-hemispheric distribution over frontocentral regions.

For the lure-1 trial, we compared the ERPs for the error-preceding, the correct-preceding, and the RT-matched correct-preceding trials. In accordance with previous reports [13], [14], the negativity immediately after the EMG onset was larger on the RT-matched correct-preceding trials than on other trials. In fact, a one-way ANOVA applied to the peak negative amplitudes at FCz showed a significant difference among these trial types (*F*(2, 36) = 5.62, *ε* = .75, *p* = .02, *pη^2^* = .24.). Post-hoc tests revealed larger negativities for the RT-matched correct-preceding trials (*M* = −2.6 µV, *SEM* = 0.51 µV) than correct-preceding trials (*M* = −1.7 µV, *SEM* = 0.48 µV), (*p* = .001) and error-preceding trials (*M* = −1.7 µV, *SEM* = 0.67 µV), (*p* = .02).

We also compared amplitudes of the negativities between correct-preceding and error-preceding trials on the five lure-preceding trials. A 2 (correct/error on lure) by 5 (pre-lure trials) ANOVA revealed that the trials prior to an error (*M* = −2.4 µV, *SEM* = 0.59 µV) had larger negative amplitudes than before correct responses (*M* = −2.0 µV, *SEM* = 0.59 µV) (*F*(1, 18) = 4.99, *p* = .04, *pη^2^* = .22). There was also a marginal effect of trial (*F*(4, 72) = 2.13, *p* = .09, *pη^2^* = .11) with larger negativities nearer to the lure although no post-hoc comparisons were significant. No interaction was found (*F*(4, 72) = 1.53, *p* = .20, *pη^2^* = .08).

Contrary to previous reports, the error-preceding positivity (EPP) was not observed. Rather, the positivity following the negative deflection was larger for correct-preceding trials than for error-preceding trials. The larger positivities for correct-preceding trials were observed even on trials preceding the lure-1 trial.

A 3 (correct/error/matched RT) by 6 (electrode sites, F3/F4/FC1/FC2/FCz/Cz) ANOVA was applied to the amplitudes of positivities on the lure-1 trial. There were no significant differences among the correct-preceding, the error-preceding, and the RT-matched correct-preceding trials (*F*(2, 36) = 2.55, *p* = .09, *pη^2^* = .12); however, because of the apparent trend a post hoc analysis was conducted but there were no significant differences among any pair of trial types. There was also a significant effect among electrode sites (*F*(5, 90) = 4.13, *ε* = .64, *p* = .009, *pη^2^* = .19), but there was no interaction (*F*(10, 180) = 1.02, *ε* = .52, *p* = .41, *pη^2^* = .05).

To clarify if the positivities are larger on the correct-preceding trials, a 2 (correct-/error-preceding) by 5 (pre-lure trials) by 6 (electrodes sites, F3/F4/FC1/FC2/FCz/Cz) ANOVA was applied to amplitudes of the positivity on the lure-1 to lure-5 trials. It revealed a significant main effect of correctness (*F*(1, 18) = 6.05, *p* = .02, *pη^2^* = .25), confirming larger positivities on the correct-preceding (*M* = 2.9 µV, *SEM* = 0.35 µV) than on the error-preceding trials (*M* = 2.5 µV, *SEM* = 0.40 µV). The effect of Electrode-site approached significance (*F*(5, 90) = 2.63, *ε* = .54, *p* = .07, *pη^2^* = .13); however, post hoc analyses failed to show any significant differences between any pair of electrodes. There was no effect of trial (*F*(4, 72) = .15, *p* = .96, *pη^2^* = .008).

**Figure 4:**
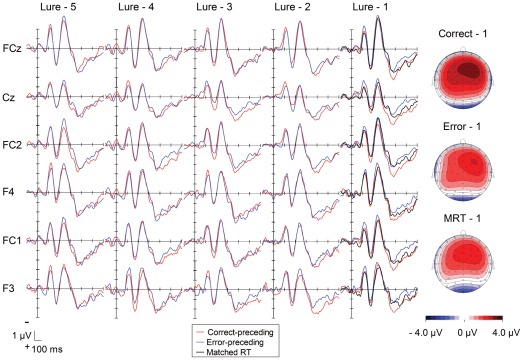
Grand-average waveforms of stimulus-locked ERPs as a function of lure preceding trials (red: correct-preceding, blue: error-preceding trials). Waveforms are drawn with negative polarity up. Scalp distributions of the positivity, ranging from 359 to 391 ms after stimulus onset, are shown for the lure-1 trials (i.e., correct-preceding, error-preceding, and matched RT trials).

Figure 4 depicts the stimulus-locked ERPs over frontocentral regions (i.e., F3, F4, FC1, FC2, FCz, and Cz) on the lure-preceding trials. The stimulus-locked ERPs showed a larger negativity peaking about 250 ms that was followed by a large positive deflection peaking about 380 ms after the stimulus presentation on both correct- and error-preceding trials. The positive deflection showed a frontocentral distribution, as was the case in the EMG-locked ERPs.

Interestingly, in accordance with the EMG-locked ERPs, the positive deflections were larger for correct-preceding trials than for error-preceding trials. In addition, a small negative deflection was observed on the descending slope of the positivity only on the error-preceding trials, suggesting a less-synchronized smeared response-related negativity that was clearly observed in the EMG-locked averaging.

A within subjects 3 (error-preceding/correct-preceding/matched-RT correct-preceding) by 6 (electrode sites, F3/F4/FC1/FC2/FCz/Cz) ANOVA was applied to the positive amplitudes on the lure-1 trial and revealed a trend for electrode site (*F*(5, 90) = 2.79, *p* = .06, *ε* = .51, *pη^2^* = .13). However, post-hoc tests showed no amplitude difference among pairs of electrode sites. There was also a main effect of trial-type (*F*(2, 36) = 4.23, *p* = .02, *pη^2^* = .19). Post hoc analyses showed a larger amplitude for correct- (*M* = 2.8 µV, *SEM* = .48 µV) than for error-preceeding (*M* = 1.9 µV, *SEM* = .49 µV) trials (*p* = .04). No interaction was observed (*F*(10, 180) = .25, *p* = .95, *pη^2^* = .01).

To clarify if the stimulus-locked positivities were larger on correct-preceding trials, a 2 (correct-/error-preceding) by 5 (pre-lure trials) by 6 (electrodes sites, F3/F4/FC1/FC2/FCz/Cz) ANOVA was applied to the amplitudes of the stimulus-locked positivity. However, it did not show any significant main effect of correctness (*F*(1, 18) = 2.23, *p* = .15, *pη^2^* = .11), trial (*F*(4, 72) = .21, *p* = .92, *pη^2^* = .01), or electrode-site (*F*(5, 90) = 1.55, *ε* = .59, *p* = .21, *pη^2^* = .08). No interaction was found (correctness by trial: *F*(4, 72) = 2.09, *p* = .09, *pη^2^* = .10; correctness by electrode-site: *F*(5, 90) = 1.82, *ε* = .57, *p* = .16, *pη^2^* = .09; trial by electrode-site: *F*(20, 360) = 1.03, *ε* = .37, *p* = .42, *pη^2^* = .05).

## Discussion

In the present study, we investigated brain activity on trials preceding correct and erroneous responses in a task with at most marginal response-conflict on these preceding trials. We found a larger early negativity in ERPs synchronized to responses preceding correctly as compared to incorrectly processed lure trials. However, contrary to previous reports, this negativity was followed by larger positivities in correct-preceding trials.

Correct responses to lure trials were slower than incorrect responses, perhaps indicating a more cautious state for correct responses. The bifurcation of RT on the lure trial relative to RT on the lure-1 trial (i.e., accelerated RT for errors and delayed RT for corrects on the lure trial) suggests that some processing was bypassed in the error responses as compared to preceding trials, resulting in speed-up, whereas for correct responses on the lure trials there may have been more involvement of inhibition, adequate stimulus-evaluation, and correction processes.

Interestingly, there were also RT differences between correct preceding and error preceding trials (i.e., on lure-4, lure-2, and lure-1 trials). The longer RTs on the correct-preceding trials relative to the overall RT suggest that correct responses to lure trials may be foreshadowed in performance as many as four trials ahead. These performance effects suggest that during the course of an experiment, the quality of stimulus processing may vary, sometimes being better, sometimes worse. Because the task used in our study involved very predictable stimuli and responses on 95% of the trials and a change of action – to lures – only on every 20^th^ trial on average, the task was likely to be very monotonous and participants may have tended to respond to the stimuli without fully processing them. In other words, because of the preponderance of stimulus/response alternations, participants may have pressed more or less automatically at alternating buttons. Such partial stimulus processing, heavily based on an automatic alternation routine, allows for fast and accurate responding as long as the expected stimulus sequence remains the same. However, if the alternation sequence is broken, the risk is high that the change is noticed only after an incorrect response has already occurred. It is also plausible that the quality of stimulus processing varies over time, for example, decreasing as a consequence of automation, and being restored at least to some extent after a break or after an error. The performance data are compatible with this idea. Errors to lures are more likely after trials that have been processed fast and automatically (error preceding RTs). Correct lure processing is more likely to have occurred after less automatized (possibly transient anticipations of a lure) and therefore somewhat slower responding (correct preceding RTs). This is supported by the overall RT that was shorter than the mean RT for the 5 correct-preceding trials, but comparable for the mean RT for the 5 error-preceding trials.

We applied a matched RT procedure to the ERP averaging on the correct lure-1 trial to compare the error-preceding and the correct-preceding ERPs following the procedure of previous studies [12], [14]. In accordance with previous findings [13], [14], our results showed a larger fronto-central negativity around 100 ms in the EMG-locked averages for the RT-matched correct-preceding than for the correct- and error-preceding (Lure-1) trials. On the other hand, a comparison of the five consecutive preceding Lure-1 to Lure-5 trials did not show significant differences in the negativities between correct-preceding and error-preceding trials. Allain et al. [13] reported that a frontocentrally distributed negativity around 100 ms was larger on the correct-preceding trials than on the error-preceding trials, suggesting performance monitoring as the functional significance of the negativity. According to this finding, it is reasonable to conclude that the larger negativity for the RT-matched correct-preceding trials in our study represented a more efficient performance monitoring, which resulted in a correct response on the lure trial.

We found a frontocentrally distributed positivity peaking about 210 ms after the EMG onset. Although previous studies have shown more positivity on the error-preceding trials and presumed that this so-called EPP reflects transient monitoring deficiencies of the ACC [12], [14], the matched RT procedure did not reveal any evidence of such an EPP in the present study. One possible reason for the failure of replication is that impairment of response monitoring did not occur in the present study because of the automatic-domain characteristic of our task. This assumption is supported by the null difference between mean RT for 5 error-preceding trials and the overall RT to lure-unrelated trials. Thus, it is possible that some asymptote of the response monitoring was responsible for the diminished EPP. Also, a study adopting a letter flanker discrimination task, in which the participants could not anticipate the forthcoming stimuli, also failed to produce an EPP [22].

By contrast to previous reports, the positivity was significantly larger on the intact (i.e., not matched-RT) correct-preceding than on the error-preceding trials. Similar results were also found in the stimulus-locked ERPs. The larger positive component on correct-preceding trials cannot be due to a different error rate on the lure trial, because our task resulted in almost a 50% error rate for the lure trials. This differs from the error rates reported on incompatible trials in response-conflict tasks; however, the error rate on the lure trials should not affect the ERPs on the preceding trials.

Because of its frontocentral scalp distribution, one may argue that the scalp distribution of the positivity is unlike the typical parietal positivity of the P300 or P3b to which such a description would fit best. However, the scalp distribution of P300 in highly predictable stimulus alternation sequences is central rather than parietal [23], [24].

Previous studies that compared error-preceding and correct-preceding trials [12], [14] did not regard the EPP as a P300-like component. In addition, the original report of the EPP conducted by Ridderinkhof et al. [12] showed much earlier latencies of the EPP (i.e., about 50 ms) than those of in the studies of Allain et al. [13] and our study (i.e., about 200 ms). The former study adopted button responses as the trigger for averaging, whereas the latter studies adopted EMG onset as a trigger, which may have resulted in different latencies of the positivity. It should also be noted that we found larger positivities even on earlier lure-preceding trials, contrary to the suggestion of Hajcak et al. [14] that a transient deficit of performance monitoring might be a specific phenomenon for trials immediately preceding the error response (i.e., the error-1 trial). Therefore, in terms of morphology and latency, it is reasonable to suggest that the positive component in our study differs from the EPP but consists of a modulation of a fronto-central P300-related component instead.

The modulations of the frontocentral positivty in our study can be interpreted also within the vigilance account suggested for the performance results. The less elaborated and more superficial processing that tends to be accompanied by incorrect lure-responses is reflected in a diminished positivity in incorrect-preceding trials than in correct-preceding trials. As in RTs this reduction of positivity is not confined to the stimulus immediately preceding the lure, in line with the idea that there may be slow changes of processing quality. The vigilance account given here is in line with the interpretation given by Eichele, Debener, Calhoun, Specht, Engel, Hugdahl, von Cramon, and Ullsperger [25] for their fMRI-data collected in a flanker task. They suggested that in addition to a decline in effortful motivated involvement there was an increase in default mode network activity many seconds prior to incorrect responses.

The present findings indicate that results previously reported regarding error-predictive ERP components do not necessarily generalize to all kinds of tasks and add to the evidence that modulations of motor-related activity foreshadow task accuracy [1], [2]. The present task, which involved at most a minimal amount of conflict yielded essentially opposite findings relative to conflict tasks. Instead of an increased positivity preceding errors as previously reported [12], we found a reduced positivity. However, we should like to point out that there are many other kinds of tasks, which might still yield other error-predictive ERP phenomena. Future research should therefore broaden the spectrum of tasks investigated to uncover the common and specific processes, predicting performance quality.

## Materials and Methods

### Participants

Nineteen female participants (mean age = 20.8, standard deviation (*SD*) = 1.7) were recruited from an undergraduate population. They had normal or corrected-to-normal vision. Eighteen were right-handed (mean handedness scores = +89.1) and one was left-handed (handedness score = −50) [26]. Informed consent was obtained from participants by their reading and signing a consent form. This study was approved by the Waseda University Academic Research Ethical Review Committee associated with the first author.

### Stimuli and apparatus

Participants were seated in a sound-attenuated, dimly lit room. Responses were recorded with two microswitch keys placed on a flat board (445×910×25 mm), 150 mm apart from each other in the participant's midsagittal plane. The participants rested both forearms and palms comfortably on the flat board to minimize any movements other than middle finger responses. Participants were instructed to place their middle fingers on the microswitch keys mounted on the board and to lift the finger of the right or left hand in a ballistic fashion when the stimulus appeared. A plastic plate (30×20×1 mm) was attached to the end of the microswitch key for the finger rest. The weight of the finger while relaxed was enough to depress the key. The displacement of the key by lifting the middle finger led to switch closure and the overt response onset could be identified. RT was measured as the interval between the stimulus onset and the microswitch closure. Stimulus presentation was produced and RT measurement was recorded by the visual-auditory stimuli presentation tachistoscope system (Iwatsu Isel, IS-702).

### Procedure and Design

We used an alternating-response task, in which a white arrowhead (pointing up or down) was presented for 200 ms with 2.0° visual angle in the center with black background on a cathode ray tube (CRT), placed 100 cm in front of the participant. Inter-stimulus interval was 600, 800, or 1000 ms, randomly selected across trials. The task was to respond to the pointing direction of the arrowhead (i.e., up or down) by briskly lifting the middle fingers (i.e., top key–up or bottom key–down). Participants were requested to respond with both speed and accuracy.

The arrowhead direction alternated for the majority of trials (95%); however, *lure* trials, where the direction of the arrowhead was the same as in the preceding trial, were presented on five percent of the trials in each block. All alternating sequences were in excess of 5 trials; thus lures were never included in any pre-lure averages. On the lure+1 trial, presentation of the stimulus sequence was reset, so that the participants were unable to anticipate the direction of the arrowhead. There were eight blocks of 200 trials each. Hand placement (left hand-distal key and right hand-proximal key, vice versa) was counter-balanced across participants.

### Recording

The electroencephalogram (EEG) was recorded from 128 sites with Ag/AgCl electrodes. Horizontal electrooculograms were recorded from the left and right outer canthi, and vertical electrooculograms from above and below the left eye. These signals were recorded with a bandwidth of DC to 205 Hz, −3 dB/octave), using the Biosemi Active Two system (Biosemi Inc.). The EMG was bipolarly recorded from the extensor digitorum muscles in the left and right forearms with Ag/AgCl electrodes using the Biosemi Active Two system, and were off-line high-pass filtered with 5.31 Hz, full-wave rectified, and low-passed filtered with 30 Hz with the Vision Analyzer (Brain Products). All physiological signals were digitized at a rate of 1024 Hz.

### Data Analysis

Processing of EEG was performed with the software package Brain Vision Analyzer (Brain Products). The EEG was re-calculated to average reference and corrected for ocular movement artifacts using the procedure described by Gratton, Coles, and Donchin [27]. According to correct and error responses on the lure trials, we classified two sequences (i.e., the correct preceding trials and the error-preceding trials). We averaged both ERPs time-locked to the EMG onset and the stimulus onset on the preceding trials for each sequence (i.e., lure-5, lure-4, lure-3, lure-2, and lure-1). Additionally, we averaged both the EMG-locked and the stimulus-locked ERPs on the lure trials and the lure+1 trial. The EMG onset was detected for each trial with a semiautomatic “macro” procedure implemented in Brain Vision Analyzer, and then was corrected by visual inspection. To determine the EMG onset, we used the criterion of a deflection of 4.0 standard deviations of the rectified EMG compared to a baseline of −700 to −500 ms pre-response using a semi-automatic macro procedure implemented in Brain Vision Analyzer. For each trial, the onset of the EMG response was determined by moving backward in time from where the upward slope of the rectified EMG waveform crossed the criterion until the amplitude ceased decreasing [28], [29]. The validity of the EMG onset detection was also visually inspected on each trial, and the invalid EMG onset was corrected manually.

For the matched RT analyses we adopted an RT-matching procedure, in which each error-preceding lure-1 trial was RT-matched to a correct preceding trial with the closest RT according to the algorithm of Hajcak et al [14].

Trials in which the RT fell outside of a 100 to 700 ms post-stimulus window or the EEG amplitude exceeded a threshold of 100 µV during the recording epoch were excluded from ERP averaging. Also excluded from the analyses were trial series that included any errors on the lure-preceding trials. ERPs were bandpass-filtered with 0.1 Hz to 30 Hz (roll-off 24 dB). The negativity in the EMG-locked ERP was measured as the largest negative peak at FCz within a window of 0 to 200 ms after the EMG onset, relative to a pre-EMG baseline (i.e., mean amplitude between −400 to −300 ms before EMG onset). This baseline was chosen because it occurs during the pre-stimulus period. Although previous studies of the EPP had used relatively few electrodes, the effect was present at fronto-central sites. This was also the case in our data. In addition we also found a right-hemispheric preponderance of the positive deflection occurring about 200 to 230 ms after EMG onset. Therefore, the positive components after EMG onset were measured as average amplitudes within a time window of 200 to 230 ms after EMG onset at F3, F4, FC1, FC2, FCz, and Cz. Here we refer to standard 10-10 electrode sites; however, the actual sites are based on the Biosemi electrode coordinate system. They are not identical but extremely close in terms of location. The stimulus-locked ERPs were scored relative to a pre-stimulus baseline (i.e., mean voltage during the 100 ms prior to stimulus onset).

RT was evaluated using a within subject, 2 by 7 ANOVA with repeated measures on correctness (correct and incorrect response on the lure trials) and trial (lure-5, lure-4, lure-3, lure-2, lure-1, lure, and lure+1). The ERP data were analyzed differently from the behavioural analysis for the reasons outlined earlier. Also, because the ERN on the lure trial should be especially elicited by error responses, the lure trial should be excluded from this analysis.

The amplitudes of the post-EMG negativity at FCz on the lure-1 trial were tested using a one-way ANOVA with repeated measures on correctness (correct-preceding, error-preceding, and RT-matched correct-preceding trial). The amplitudes of the post-EMG positivity on the lure-1 trial were tested using a within subject 3 by 6 ANOVA with repeated measures on correctness (correct-preceding, error-preceding, and RT-matched correct-preceding trial) and electrode sites (F3/F4/FC1/FC2/FCz/Cz). We scored amplitudes over these electrodes, because the positivity showed a broad but slightly right-frontocentral distribution (see results section).

To investigate the time-course effect on the ERPs, the amplitudes of the post-EMG positivity were also analyzed using a 2 by 5 by 6 ANOVA on correctness (correct-preceding and error-preceding), preceding-trial (lure-5, lure-4, lure-3, lure-2, and lure-1), and electrode site (F3/F4/FC1/FC2/FCz/Cz). Where post hoc comparisons were required, the Bonferroni correction was applied. We reported the Greenhouse-Geisser epsilon value along with the original degrees of freedom and if the assumption of sphericity was violated the adjusted significance level (*p* value).
